# Recurrent Cancer-Associated Nonbacterial Endocarditis Presenting With Systemic Embolic Complications

**DOI:** 10.1016/j.jaccas.2025.105161

**Published:** 2025-09-24

**Authors:** Kanna Nakamura, Yugo Yamashita, Shingo Koyama, Masumi Sunada, Masahiro Tanji, Takahiro Horie, Koh Ono

**Affiliations:** a Department of Cardiovascular Medicine, Graduate School of Medicine, Kyoto University, Kyoto, Japan; b Department of Obstetrics and Gynecology, Graduate School of Medicine, Kyoto University, Kyoto, Japan; c Department of Neurosurgery, Graduate School of Medicine, Kyoto University, Kyoto, Japan

**Keywords:** cancer-associated thrombosis, nonbacterial thrombotic endocarditis, ovarian cancer, transthoracic echocardiography, transesophageal echocardiography

## Abstract

**Background:**

Nonbacterial thrombotic endocarditis (NBTE) is a rare complication of malignancy, often leading to systemic embolization.

**Case Summary:**

A 52-year-old woman presented with embolic ischemic stroke, multiple systemic infarctions, a pelvic mass, and aortic valve vegetations. The pelvic mass was completely resected and was subsequently diagnosed as ovarian clear cell carcinoma. The valvular vegetations were identified as NBTE. Despite ongoing anticoagulation therapy with edoxaban, she developed recurrent vegetations on the mitral valves in association with cancer recurrence. Based on the patient's preference, anticoagulation therapy with edoxaban was continued. She later experienced an upper limb arterial embolism and died after 3 months.

**Discussion:**

This case highlights the diagnostic and therapeutic challenges of NBTE in cancer patients, particularly in the context of temporally distinct recurrences involving different cardiac valves despite appropriate oncologic and anticoagulant therapy.

**Take-Home Message:**

Vigilant valvular monitoring and individualized anticoagulation strategies are necessary when managing patients with hypercoagulable malignancies.

## Visual Summary

**Figure:**
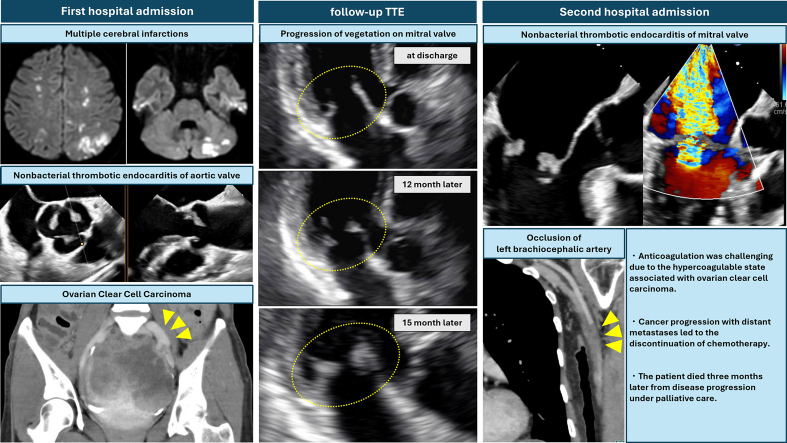

## History of Presentation

A 52-year-old woman presented to the emergency department with sudden-onset right-sided hemiparesis and dysarthria. Her vital signs included blood pressure of 124/82 mm Hg, pulse rate of 90 beats/min, respiratory rate of 15 breaths/min, temperature of 37.0 °C, and oxygen saturation level of 99%. Neurological examination revealed cerebellar ataxia of the right upper and lower limbs, right homonymous hemianopsia, and right spatial neglect. The patient's National Institutes of Health Stroke Scale score was 7 at presentation. She had no chest pain, dyspnea, or recent infections.

**Take-Home Messages**

• NBTE is a potential cause of multiple embolic events and progressive valvular dysfunction, particularly in patients with a hypercoagulable state due to occult or known malignancy.
• NBTE can involve different cardiac valves at different time points, highlighting the importance of comprehensive and repeated valve assessment in high-risk patients.

**Figure 1:**
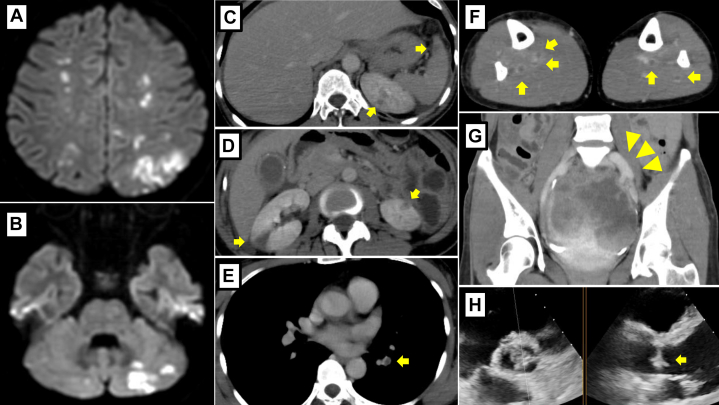
Initial Diagnostic Findings (A and B) Multiple cerebral infarctions identified on brain MRI. (C and D) Splenic infarction and multiple renal infarctions observed on contrast-enhanced CT (arrows). (E) Pulmonary embolism in the lower lobe of the left lung (arrow). (F) Multiple deep vein thromboses (arrows). (G) A large contrast-enhanced pelvic mass (arrowheads). (H) Mobile vegetations on aortic valve detected by TTE (arrow). CT = computed tomography; MRI = magnetic resonance imaging; TTE = transthoracic echocardiography.

Brain magnetic resonance imaging showed multiple cerebral infarctions suggestive of a cardioembolic origin, without any infected aneurysm (Figures 1A and 1B). In addition, contrast-enhanced whole-body computed tomography (CT) revealed multiple infarcts, including pulmonary embolism, deep vein thrombosis, splenic and renal infarctions, and a large pelvic lesion (Figures 1C to 1G). Electrocardiogram showed sinus rhythm. Chest x-ray showed no cardiomegaly or pulmonary congestion. Transthoracic echocardiography (TTE) revealed a small mobile mass on all 3 coronary cusps of the aortic valve (Figure 1H). Blood tests showed anemia and abnormal coagulation parameters, with a hemoglobin level of 9.9 g/dL and a markedly elevated D-dimer level of 71.3 μg/mL. Inflammatory markers and white blood cell count were within limits of normal range.

## Past Medical History

The patient had no past medical history of prior thromboembolic events, cardiovascular disease, or malignancy. She was a nonsmoker and had no family history of cancer or thrombophilia.

## Differential Diagnosis

The differential diagnosis of ischemic stroke includes large artery atherosclerosis, small vessel occlusion, and cardioembolic stroke, often due to atrial fibrillation, recent myocardial infarction, or structural cardiac abnormalities. When a cardiac source is suspected, the differential diagnoses include vegetations (infective or nonbacterial thrombotic endocarditis [NBTE]), Libman-Sacks endocarditis, and antiphospholipid syndrome, as well as cardiac tumors such as papillary fibroelastoma, Lambl excrescences, or myxoma. In cryptogenic stroke, other possibilities include paradoxical embolism through a patent foramen oval as well as noncardiac embolic sources such as artery-to-artery embolism and cancer-associated hypercoagulability.

## Investigation

**Figure 2:**
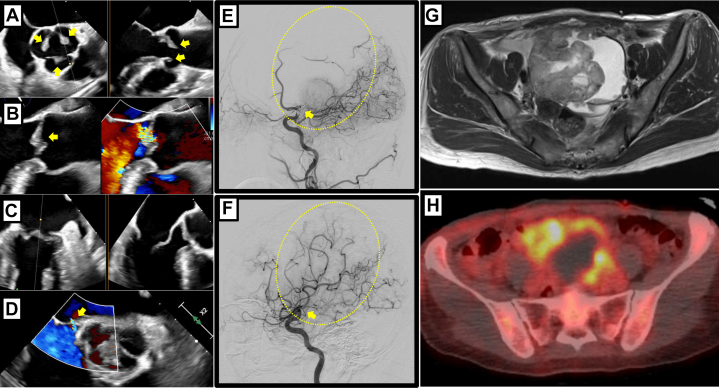
Imaging Before Tumor Resection (A and B) Mobile vegetations on all of the aortic valves (arrows) and mild aortic regurgitation (arrow) by TEE; the largest vegetation measured 7 × 6 mm on the left coronary cusp. (C) No vegetations were detected at the mitral valve. (D) A small atrial septal defect was found (arrow). (E and F) Occlusion and recanalization of the left middle cerebral artery demonstrated on sagittal view before and after the procedure (arrows). (G) MRI showing a solid mass with a maximum diameter of 9.2 cm, with contrast enhancement, diffusion restriction, and expansile features in the pelvic cavity. The origin of the lesion is presumed to be the left ovary. The cystic component showed mildly high signal intensity on T1-weighted imaging. (H) PET/CT demonstrated an irregular cystic mass compressing the uterus to the right side in the midline of the pelvis. Within the lesion, an area of thickened solid wall demonstrated intense and heterogeneous uptake of fluorine-18-fluorodeoxyglucose. There was no definitive evidence of distant metastasis. MRI = magnetic resonance imaging; PET/CT = positron emission tomography/computed tomography; TEE = transesophageal echocardiography.

After hospitalization, transesophageal echocardiography TEE showed vegetations on all 3 coronary cusps of the aortic valve and a small atrial septal defect. No vegetation was found on the other valves (Figures 2A to 2D). Tumor markers were also elevated: carbohydrate antigen-125 was 395 U/mL, and neuron-specific enolase was 26.4 ng/mL. No protein C or protein S, anti-β2 glycoprotein I antibodies, anticardiolipin antibody, antinuclear antibody, or lupus anticoagulant were detected in plasma.

## Management

Unfractionated heparin was initiated immediately, and vancomycin and ceftriaxone were administered to the patient given a possibility of infective endocarditis that could not be ruled out. The following day, the activated partial prothrombin time lengthened, from 43.4 seconds at admission to 51.5 seconds, although her paralysis showed improvement. However, the right-sided paralysis reappeared owing to occlusion of the left middle cerebral artery. Reperfusion therapy was successfully achieved through catheter-directed mechanical thrombectomy; however, the right-sided paralysis persisted (Figures 2E and 2F).

**Figure 3:**
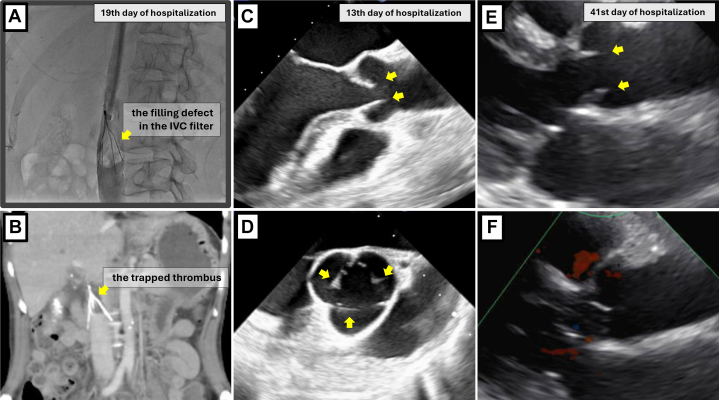
Imaging After Tumor Resection (A and B) Attempted removal of the inferior vena cava (IVC) filter using a catheter was unsuccessful because of a trapped thrombus, seen as a filling defect. (C and D) Regression of aortic valve vegetations (arrows) observed intraoperatively by TEE on the 13th day of hospitalization. (E and F) Complete resolution of all 3 aortic valve vegetations (arrows) by TTE at hospital discharge on the 41st day of hospitalization. TEE = transesophageal echocardiography; TTE = transthoracic echocardiography.

Magnetic resonance imaging showed a solid mass with contrast enhancement, diffusion restriction, and expansile features in the pelvic cavity (Figure 2G). Positron emission tomography/CT demonstrated uptake of fluorine-18-fluorodeoxyglucose in the mass (Figure 2H). The pelvic mass, suspected of being an ovarian malignancy and associated with a hypercoagulable state, prompted tumor debulking surgery after inferior vena cava filter placement on day 14 of hospitalization. The tumor was completely resected, and histopathological examination confirmed the mass as ovarian clear cell carcinoma (OCCC). After surgery, the inferior vena cava filter could not be removed owing to thrombus formation and was left in place permanently (Figures 3A and 3B).

Postoperative anticoagulation therapy was initiated with apixaban 10 mg daily; however, genital bleeding occurred, and the regimen was switched to edoxaban 30 mg daily. TEE performed during the surgery and TTE performed 1 month postoperatively demonstrated complete resolution of the cardiac findings (Figures 3C to 3F, Video 1). Three sets of blood cultures were negative, supporting a diagnosis of NBTE.

**Figure 4:**
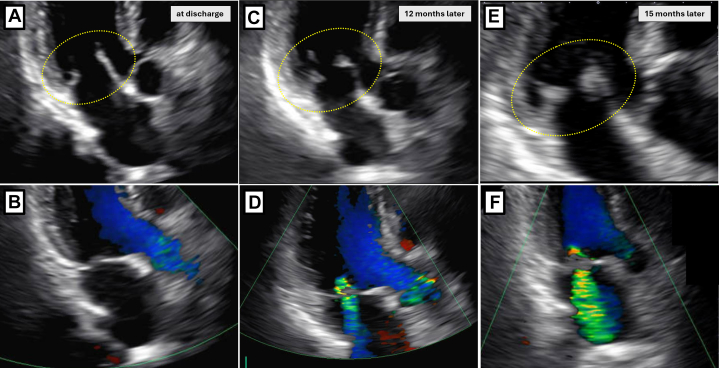
Progression of Mitral Valve Vegetations on TTE (A and B) At discharge, no vegetations were observed on the mitral leaflets, and no mitral regurgitation was present. (C and D) At 12 months after discharge, thickening of the mitral valve leaflets appeared, accompanied by mild-to-moderate mitral regurgitation. (E and F) At 15 months after discharge, the vegetations had enlarged, and severe mitral regurgitation developed. TTE = transthoracic echocardiography.

**Figure 5:**
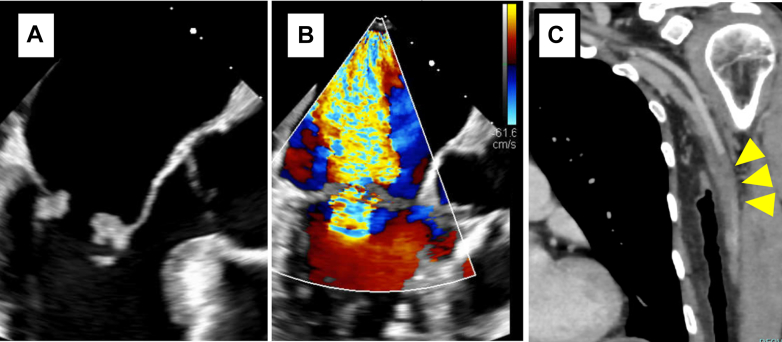
TEE and Contrast-Enhanced CT Findings After Recurrent NBTE (A and B) TEE revealed vegetations on both mitral valve leaflets, with the largest measuring 9 × 7 mm on the anterior leaflet. These findings were accompanied by severe mitral regurgitation. (C) Contrast-enhanced CT demonstrated occlusion of the left brachiocephalic artery (arrowheads). CT = computed tomography; NBTE = nonbacterial thrombotic endocarditis; TEE = transesophageal echocardiography.

The patient remained stable for approximately 12 months without recurrence of cancer or embolic events. However, follow-up contrast-enhanced CT revealed multiple peritoneal disseminations indicating cancer recurrence. Chemotherapy with paclitaxel, gemcitabine, and carboplatin was initiated. Her hemoglobin dropped to 6.6 g/dL at one point, prompting blood transfusion. She remained compliant with edoxaban 30 mg. TTE and TEE performed during this period revealed progressive mitral regurgitation with vegetations on both mitral valve leaflets, which had advanced to severe mitral regurgitation (Figures 4E and 4F, 5A and 5B, Video 2). Retrospective review of the earlier TTE images obtained around 12 months postdischarge revealed subtle thickening of the mitral valve leaflets and progression of mitral regurgitation that had not been recognized at the time, suggesting the early development of NBTE-related changes (Figures 4A to 4D). The patient was admitted to the hospital, and empirical intravenous administration of vancomycin and ceftriaxone was reinitiated owing to suspicion of vegetations suggestive of infective endocarditis. Oral edoxaban 30 mg was continued as anticoagulation therapy, based on the patient's preference. At the time of admission, her hemoglobin level was 8.8 g/dL and D-dimer level was 12.6 μg/mL, and a transfusion was required. Although the N-terminal pro–B-type natriuretic peptide level was elevated at 652 pg/mL, there was no clinical evidence of decompensated heart failure. On day 14 of hospitalization, her left palm suddenly became pale and pulselessness on the radial artery. Contrast-enhanced CT demonstrated a brachial artery embolism (Figure 5C). As there were no sensory or motor deficits, and her creatinine-kinase levels were not elevated, the patient opted for conservative management. The embolic mass was diagnosed as recurrence of NBTE with persistently negative blood cultures. Antibiotic therapy was discontinued after the diagnosis.

## Follow-Up

The discoloration of the patient's fingers gradually improved. However, the cancer progressed with the development of distant metastases, leading to the discontinuation of chemotherapy. Advance care planning was conducted, and her care was transitioned entirely to palliative management. She was then transferred to a palliative care hospital, and she died 3 months later from progression of the cancer.

## Discussion

This case presented an unusual clinical course and could provide clinically relevant insight for cancer-associated NBTE. First, NBTE preceded the diagnosis of OCCC. Second, NBTE recurred at different times and involved different valves, despite guideline-adherent oncologic and anticoagulation management. This underscores a critical gap in our current understanding of thromboembolic risk and management in hypercoagulable malignancies and calls for monitoring strategies in high-risk patients.

OCCC is a rare subtype of epithelial ovarian cancer, accounting for 5% to 11% of cases.^1^ Although frequently diagnosed at an early stage, it carries a poor prognosis in advanced stages and is resistant to platinum-based chemotherapy.^2^ Complete cytoreductive surgery is the standard treatment, with no adjuvant chemotherapy recommended after comprehensive surgical staging.^3^ Patients with OCCC have a 2.5-fold higher risk of venous thromboembolism compared with other subtypes, making thromboembolism a critical prognostic factor.^4^

NBTE is characterized by sterile vegetations composed of fibrin and platelet on cardiac valves.^5^ It is uncommon, with an incidence of 1.1% to 1.6% in autopsy studies.^6^ Approximately 41% of NBTE cases are associated with malignancy.^7^ Stroke is the most frequent clinical manifestation, occurring in approximately 60% of patients. The mitral valve is more often involved than the aortic valve, and TTE is used to confirm the diagnosis in approximately 45% of cases.^8^ Managing the underlying malignancy is key to preventing recurrence. Anticoagulation therapy is generally recommended.^9^^,^^10^

In our patient, NBTE recurred despite early detection of OCCC and appropriate oncologic treatment. Anticoagulation management was challenging owing to transfusion-dependent anemia and the hypercoagulable state associated with OCCC. Although evidence supporting the efficacy of direct oral anticoagulants in NBTE is limited, edoxaban was chosen because of the coexisting venous thromboembolism. Notably, progressive valvular dysfunction of unclear origin served as a clinical clue for recurrent NBTE.

This case highlights the importance of vigilant cardiac monitoring and individualized anticoagulation strategies in patients with malignancy-associated thromboembolic disorders. NBTE should be considered in the differential diagnosis of unexplained progressive valvular dysfunction, especially in patients with cancer. Furthermore, the efficacy of direct oral anticoagulants in the management of NBTE remains uncertain and warrants further investigation.

Previous case reports have described NBTE affecting multiple cardiac valves simultaneously in the setting of advanced malignancy. Bagheri et al^11^ reported NBTE involving the aortic, mitral, and tricuspid valves concurrently in a patient with metastatic colorectal carcinoma. Rankin et al^12^ described involvement of the aortic and mitral valves in a patient with relapsed acute myeloid leukemia. In contrast, to our knowledge, no prior cases have described recurrence of NBTE at different time points and involving different cardiac valves. Our case is therefore unique in demonstrating temporally distinct NBTE episodes affecting separate valves—initially the aortic valve, and later the mitral valve—despite adherence to guideline-based oncologic and anticoagulant therapy.

## Conclusions

This case highlights an unusual clinical course of recurrent NBTE in patients with OCCC despite the appropriate management of cancer and anticoagulation therapy. It should be noted by clinicians that NBTE could manifest as unexplained progressive valvular dysfunction initially and be the underlying cause of multiple embolic events, leading to the diagnosis of occult cancer with OCCC.

**Visual Summary:**
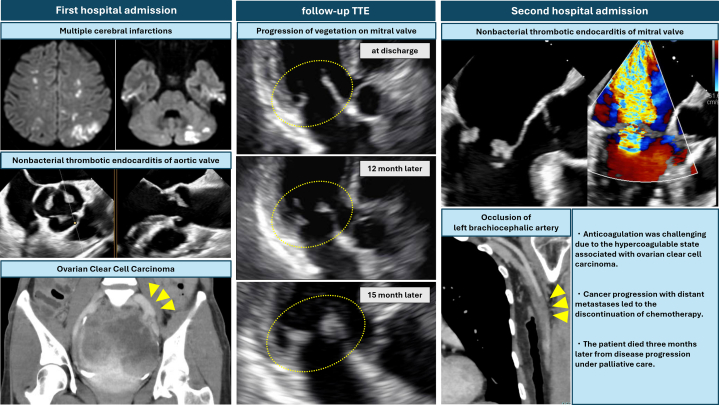
Recurrent NBTE With Systemic Embolic Events TTE = transthoracic echocardiography.

## Funding Support and Author Disclosures

The authors have reported that they have no relationships relevant to the contents of this paper to disclose.

## Appendix

Video 1 Transesophageal Echocardiography Demonstrates Mobile Vegetations on All Aortic Valve Cusps

Video 2 Transesophageal Echocardiography Reveals Vegetations on Both Mitral Valve Leaflets With Severe Mitral Regurgitation
